# Case-report: Massive infection by *Cysticercus longicollis* in a captive *Lemur catta* from Italy

**DOI:** 10.3389/fvets.2023.1288451

**Published:** 2023-11-06

**Authors:** Matteo Cuccato, Selene Rubiola, Luca Rossi, Sara Piga, Frine Eleonora Scaglione

**Affiliations:** ^1^Department of Veterinary Sciences, University of Turin, Turin, Italy; ^2^Fondazione Zoom, Turin, Italy

**Keywords:** *Lemur catta*, *Taenia crassiceps*, zoo, ring-tailed lemur, Italy

## Abstract

An adult male ring-tailed lemur (*Lemur catta*) from a biopark of northern Italy was submitted to necropsy. A multi-organ parasitic infection was macroscopically evident. Abundant sero-hemorrhagic fluid with larval parasites was present in all cavities. The microscopic evaluation of parasites and the molecular characterization revealed the presence of *Cysticercus longicollis* (the larval stage of *Taenia crassiceps*). Histology of liver, lungs, intestine and urinary bladder revealed several larval parasites surrounded by a severe lymphocytic infiltrate, fibrous tissue and hemorrhages. This is the first report of a ring-tailed lemur with an infection of *C. longicollis* in Italy. The source of infection is still not known however, the discovery of this parasite in a captive lemur poses more attention on the control of parasitic diseases implementing monitoring tests and biosecurity measures.

## 1. Introduction

In the past 20 years, the occurrence of parasitic diseases in captive *Lemur catta* has been largely reported. The ring-tailed lemur is an endangered non-human primate native to Madagascar and its natural habitat is still strictly related only to that country (1). However, this species easily adapts in zoos and wildlife parks worldwide, allowing many conservational programs to preserve this primate species. In this context, an appropriate medical monitoring of captive animals is a fundamental activity in zoo management. Special attention is given to parasitic diseases, in particular as a source of zoonotic threat. Less is known about parasites inhabiting lemurs as definitive hosts, and the only available information are just related to case-reports published in scientific literature (2–6). In only four case-reports are described severe infections in ring-tailed lemurs by *Echinoccous multilocularis* in Japan and France (7, 8) and by *Echinoccous equinus* and *Echinoccous ortleppi* in the UK (9, 10). However, recent findings suggest the presence of an emerging cestode parasite, i.e., *Taenia crassiceps* and its larval form *Cysticercus longicollis*, affecting ring-tailed lemurs in Europe with reported cases in Spain (11), Bosnia Erzegovina (12), Poland (13), and Serbia (14). Moreover, this parasite was also described in a black lemur (*Eulemur macaco macaco*) (15). *Taenia crassiceps* is a cestode parasite with an indirect life cycle, classically found in the northern hemisphere of the world (12). The definitive hosts are wild carnivores, in particular red foxes (*Vulpes vulpes*), artic foxes (*Vulpes lagopus*), and wolves (*Canis lupus*), but sometimes also domestic dogs (*Canis lupus familiaris*) (16). On the other hand, intermediate hosts are wild rodents, which are natural preys of the definitive hosts (16). In carnivores, *T. crassiceps* has a small intestinal localization and proglottids are released with feces (16). In this way, intermediate hosts can develop the larval stage of the disease after the ingestion of proglottids. In rodents, normally *C. longicollis* has a subcutaneous localization and sometimes also within body cavities (16). In addition, *T. crassiceps* is also a zoonotic parasite and primates can become intermediate hosts trough the consumption of contaminated food or water (14). In human, the disease is mainly described in immunocompromised patients with subcutaneous and muscle cysticercosis (17). As already described, also non-human primates can be susceptible to *C. longicollis* infection suggesting an emerging zoonotic role for this parasite. In this context, the main aim of this case-report study is to describe the case presentation and diagnostic assessment of the first reported observation of *T. crassiceps* cysticercosis in a captive ring-tailed lemur (*L. catta*) in Italy.

## 2. Case description

In May 2022, an adult male ring-tailed lemur (*L. catta*) from a biopark of northern Italy was submitted to necropsy at the Department of Veterinary Sciences in Turin. The animal was living with other lemurs and was donated in 2012 from a zoo in Sicily. In the park, lemurs were living on an artificial island without any possibility of contact with domestic or wild canids. Before its death, the lemur was the only one in the group with a swollen abdomen, but with normal appetite and general condition. After induction and mask maintenance with isoflurane dose-effect, the animal was subject to clinical examination and abdominal echography revealing the presence of a severe abdominal ascites, with the presence of severe abdominal effusion and of a 5 cm diameter cyst in abdomen. Therefore, the abdominal fluid collected by ultrasound-guided cystocentesis was characterized by citric color and full of small white particles, which were examined at light microscopic confirming the presence of parasites infection. The day after, the animal was subjected to an explorative laparotomy with the aim of removing the cyst and performing an abdominal lavage. However, due to the too severe clinical presentation with massive parasitic infection the animal was euthanized. The remaining lemurs of the group were subjected to clinical examination and echography excluding other infested cases, nevertheless all animals were still treated with a therapeutic protocol of albendazole (10 mg/kg once a day for 3 consecutive days and repeated with same protocol after 2 weeks).

## 3. Diagnostic assessment

**Figure 1:**
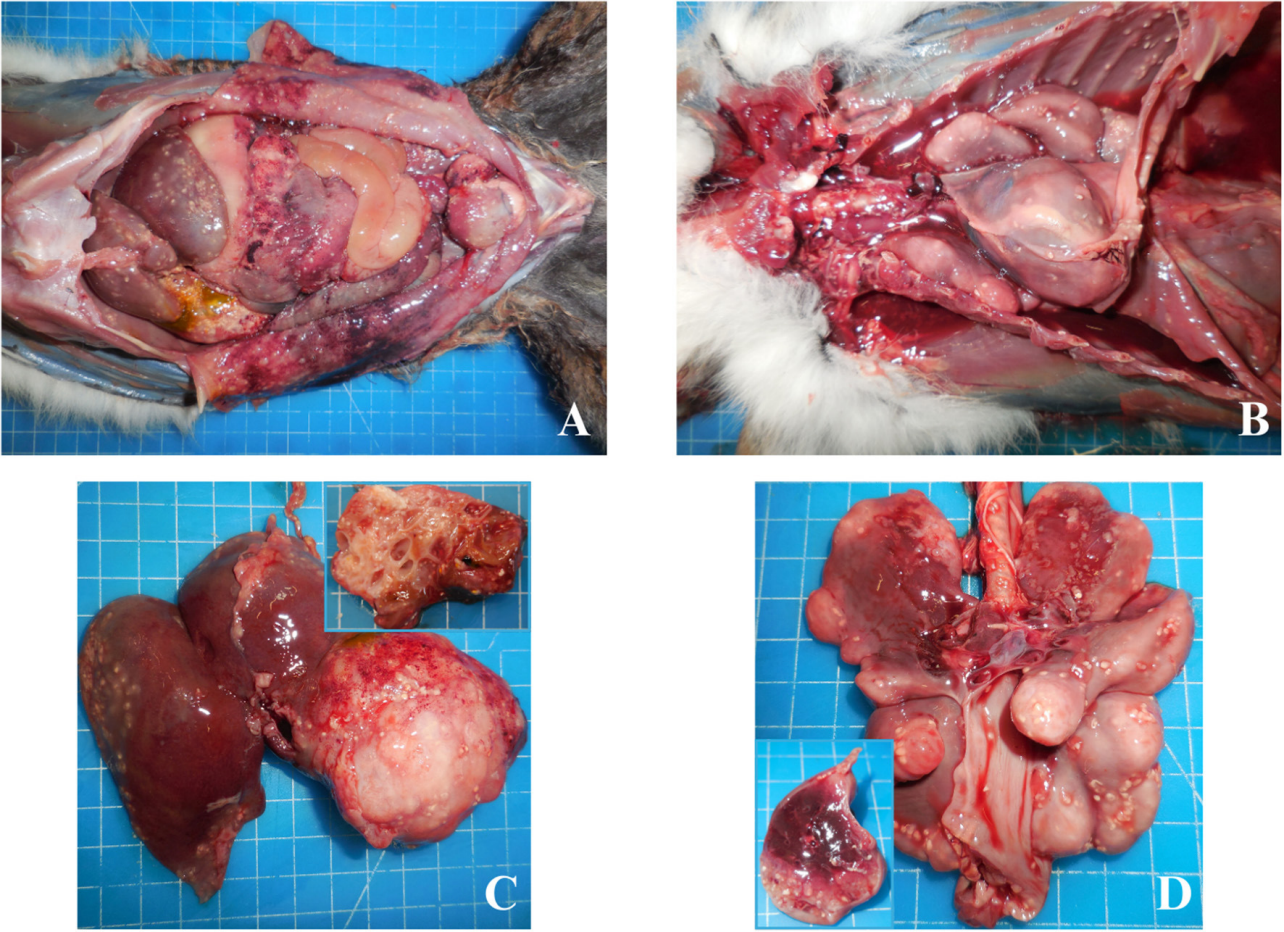
*Lemur catta*; adbomen, torax, liver and lungs. Abundant sero-hemorrhagic fluid with larval parasites was present in the abdomen **(A)** and thorax **(B)**. Enlarged liver with cyst **(C)**, composed of a multilocular structure (insert). Lungs with cystic lesions **(D)**, augmented consistency and with a multilocular structure (insert).

At necropsy, a multi-organ parasitic infection was macroscopically evident. Abundant sero-hemorrhagic fluid with larval parasites was present both in abdomen (Figure 1A) and thorax (Figure 1B). The liver, the main organ presenting lesions, was enlarged with a 5 cm diameter cyst in the parenchyma with a multilocular structure, filled with citrine fluid and larval parasites (Figure 1C). Cystic lesions were bilaterally also found in the lungs, with variable dimensions from 1 to 3 cm, augmented consistency and with a multilocular structure (Figure 1D). The presence of peritonitis, pleuritis and pericarditis was also recorded, together with the presence of disseminated larval parasites in all serosae. Kidneys and the remaining organs had no evident macroscopic lesions. Samples for histological, parasitological, and biomolecular investigations were taken and appropriately stored. Liver, kidney, seminal vesicles, urinary bladder, intercostal skeletal muscle, lung, heart and spleen were collected and fixed in 4% buffered formalin. The abdominal wall, spleen, kidney, lung, liver and seminal vesicles were also collected and immediately frozen at −20°C for microbiological and parasitological investigations. In addition, feces were collected from rectum for parasitological investigations.

**Figure 2:**
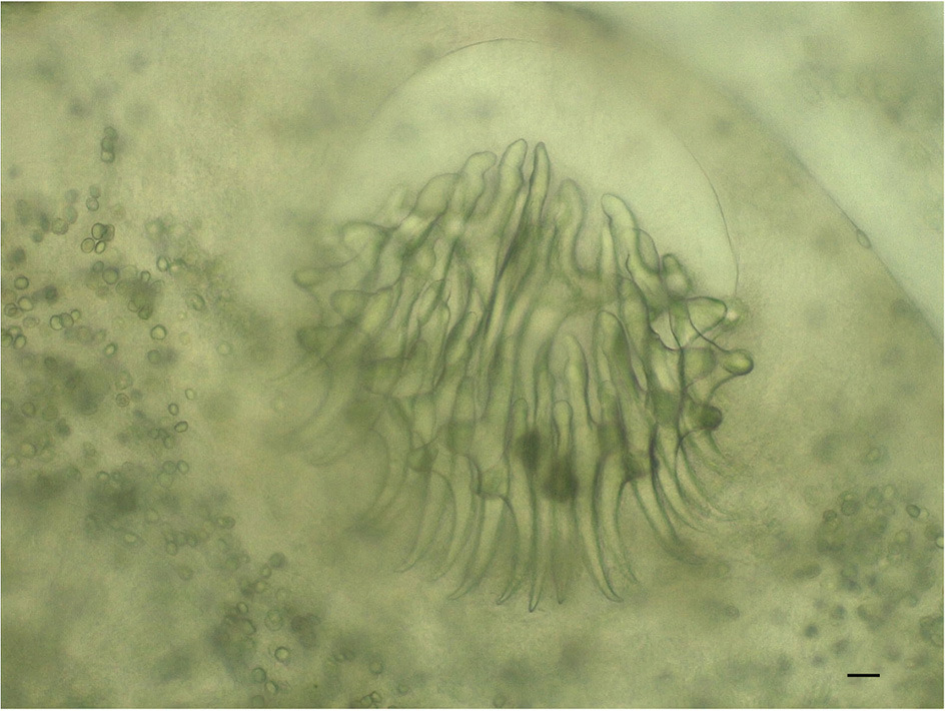
*Taenia crassiceps*. Scolex with hooks of the cysticercus, isolated from liver (200×, bar 500 μm).

The microscopic observation of parasites was consistent with *T. crassiceps* cysticerci (Figure 2). In particular, in line with literature (18–20), we emphasize: (i) the high number of ovoid to elongate cysticerci, several hundred in number; (ii) the variable size of cysticerci, with the largest ones ranging between 2–7 mm in length and 1–3 mm in width; (iii) in some of the larger cysticerci, the occurrence of endogenous and/or exogenous budding at the end opposite the invaginated scolex; (iv) the number of rostellar hooks, in the range of 30–34/scolex, including a similar number of small and large hooks; (v) the length of fully developed hooks, in the order of 150 and 120 μm for large and small hooks, respectively, and their shape, with the blade appearing much longer than the handle (approximately the double).

DNA was extracted from two cysticerci using DNeasy Blood and Tissue Kit (Qiagen), according to the manufacturer's tissue protocol; the lysis step was carried out at 56°C overnight with Proteinase K. The PCR protocol described by Bowles et al., targeting the cytochrome C oxidase subunit I mitochondrial (mtDNA cox1) gene was conducted on extracted DNA (21). The PCR mixture contained 2.5 μl of template DNA (5–20 ng/μl), 0.5 mM of each primer (JB3 TTTTTTGGGCATCCTGAGGTTTAT and JB4.5 TAAAGAAAGAACATAATGAAAATG), 2 mM MgCl_2_, 0.2 mM of each dNTP, 1 U Platinum Taq DNA polymerase, 10× PCR buffer and RNase-free water to a total volume of 25 μl. The amplification was performed in an Applied Biosystems 2720 Thermal Cycler (Applied Biosystems, CA, USA) with the following cycling profile: a denaturation step at 94°C for 5 min, followed by 30 cycles at 94°C for 40 s, 55°C for 30 s and 72°C for 30 s and final extension 72°C for 5 min. Positive results were observed with an agarose gel electrophoresis. Subsequently, amplicons were sequenced to confirm parasite species. Amplification products were purified with Exo-Sap (USB Europe, Staufen, Germany) treatment according to the manufacturer's recommendations. Forward and reverse sequencing reactions were performed using ABI Prism BigDye Terminator Cycle Sequencing Ready Reaction Kit, version 1.1 (Applied Biosystems, Foster City, CA). Sequenced fragments were purified by DyeEX (Qiagen, Hilden, Germany) and resolved by capillary electrophoresis using an ABI 310 Genetic Analyser (Applied Biosystems, Foster City, CA). Forward and reverse sequences were manually assembled into consensus sequences using the Alignment Explorer within MEGA X (22).The nucleotide sequences were analyzed using the BLASTN sequence similarity search at the NCBI database. The sequencing of the amplified products resulted in two sequences of 433 bp each, with 100% identity with published sequences *T. crassiceps* (accession number KY321321). Sequences generated in the present study were submitted in GenBank under accession numbers OR350515 and OR350516. Fecal samples for the case and for all the other lemurs in the same environment were analyzed with flotation technique for parasitological diagnosis (23). Samples tested negative for any intestinal parasites.

**Figure 3:**
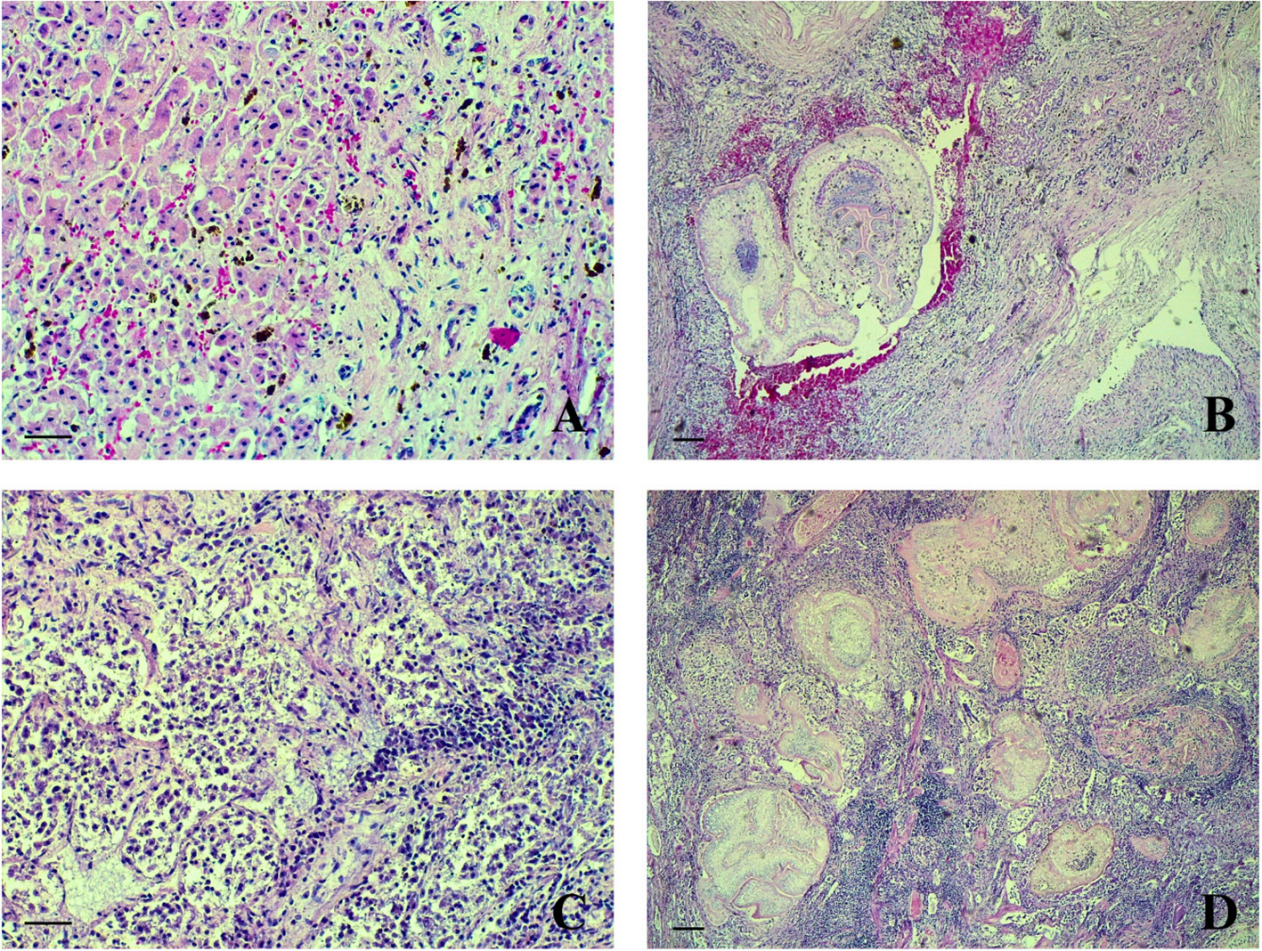
*Lemur catta*, liver, lungs. Lymphocytic infiltrate, fibrous tissue and hemorrhages in the liver **(A)** and lungs **(C)** (HE, 200×, bar 500 μm). Larval parasites in the parenchyma of liver **(B)** and lungs **(D)** (HE, 40×, bar 1,000 μm).

Histology of liver revealed several larval parasites surrounded by a severe lymphocytic infiltrate, fibrous tissue, hemosiderosis and hemorrhages (Figures 3A, B). Lungs (Figures 3C, D) presented inflammatory lesions similar to those observed in the liver. Moreover, moderate emphysema, oedema and focal necrosis were observed. Intestine was hyperemic with focal hemorrhages in the muscular layer and inflammatory lesions associated to parasites, similar to the abovementioned organs, were also observed. Pleura and peritoneum were thickened, and severe lymphocytic infiltrates were also present. In the urinary bladder severe congestion and perivascular lymphocytic infiltrate with fibrous tissue and hemorrhages were observed. Spleen presented severe lymphocytic depletion and diffuse subcapsular hemorrhages. The intercostal skeletal muscle was characterized by parasitic cysts surrounded by a severe lymphocytic infiltrate, fibrous tissue and hemorrhages both in the epimysium and within the muscular fibers. Kidney and heart did not present any histological lesions.

Finally, microbiological investigations have been conducted by an external laboratory. The presence of the main non-human primate pathogens has been investigated in organs collected during necropsy. In particular, Simian immunodeficiency virus (SIV), Simian Type D Retrovirus (SRV) and Herpesvirus of exotic animals (HVES), but also of *Neospora caninum* and *Toxoplasma gondii* have been investigated by PCR or sequencing (for HVES) according to the external laboratory routine methods. Moreover, bacteriological culture was performed through non-selective conditions according to the external laboratory routine methods. All microbiological tests resulted negative for the research in all collected organs of the abovementioned pathogens.

## 4. Discussion

According to previous scientific literature, this is the first report of a ring-tailed lemur with an infection of *C. longicollis* in Italy. Case presentation and related findings were similar to those already reported in other European countries (Spain, Bosnia Herzegovina, Poland and Serbia) (11–14). Therefore, this study represents the fifth published case report of a *T. crassiceps* cysticercosis in a captive ring-tailed lemur (*L. catta*) worldwide.

Considering that lemurs were living on an artificial island without any possible contact with domestic or wild canids, the most likely route of infection of this atypical intermediate host is contaminated water or feed. Nevertheless, the proliferative nature of *T. crassipes* cysticercosis deserves caution in ruling out other possible sources, as suspected in humans (17). Although the source of infection is still unknown, the discovery of this parasite in a captive lemur poses more attention on the control of parasitic diseases by implementing monitoring tests and biosecurity measures.

In the present case, the lemur did not present a subcutaneous infection, which is typical of intermediate and paratenic hosts. In fact, macroscopic cutaneous lesions were not observed, as reported in previous case reports (11, 13, 14). The massive infection found involving all body cavities and organs is similar to the case report of Alić et al. (12). These findings may suggest a correlation with an immunocompromised status, as described in humans (24, 25), and also in carnivores (20), where patients were diagnosed with an immune deficiency syndrome or HIV positive. Even if an immune deficiency of the lemur during life could not be excluded, the subject tested negative for bacteriological and virological (SIV and SRV) diseases. Further studies have to be conducted to evaluate the invasive and aggressive nature of this parasite in lemurs.

The negative results at parasitological investigation prove that the lemur was not eliminating the parasite through feces confirming its role as intermediate host.

## 5. Conclusions

The prevalence of *C. longicollis* in *L. catta* was never investigated worldwide, moreover, the prevalence of the parasite is not known also in intermediate hosts belonging to wild rodent species. In recent years, epidemiological investigation has been conducted in Italy on wild wolves and red foxes reporting some positive results for *T. crassiceps* in these species and confirming the presence and dissemination of the parasite in the area (26, 27).

In conclusion, further studies are need for a better understanding of *T. crassiceps* prevalence in definite and intermediate hosts in Italy. In addition, epidemiological studies evaluating the role of rodents as intermediate hosts of the parasite and their relationship with captive animals are needed. In this way, a better clarification of the epidemiological aspects of *T. crassiceps* will allow an optimal implementation of prophylactic and biosecurity measures in zoos and wildlife parks.

## Data availability statement

The original contributions presented in the study are publicly available. This data can be found here: https://www.ncbi.nlm.nih.gov/genbank/, OR350515-OR350516.

## Ethics statement

Ethical approval was not required for the study involving animals in accordance with the local legislation and institutional requirements because the lemur was submitted to necropsy after its death as a regular procedure of the zoo. This action did not require an ethical approval.

## Author contributions

MC: Conceptualization, Data curation, Formal analysis, Investigation, Methodology, Visualization, Writing – original draft. SR: Data curation, Formal analysis, Investigation, Methodology, Writing – review & editing. LR: Methodology, Writing – review & editing. SP: Methodology, Resources, Writing – review & editing. FS: Conceptualization, Data curation, Investigation, Methodology, Project administration, Resources, Supervision, Writing – review & editing.
